# Validation of natural language processing methods capturing semantic incoherence in the speech of patients with non-affective psychosis

**DOI:** 10.3389/fpsyt.2023.1208856

**Published:** 2023-07-25

**Authors:** Sandra Anna Just, Anna-Lena Bröcker, Galina Ryazanskaya, Ivan Nenchev, Maria Schneider, Felix Bermpohl, Andreas Heinz, Christiane Montag

**Affiliations:** ^1^Department of Psychiatry and Neurosciences, Campus Charité Mitte, Charité – Universitätsmedizin Berlin, Corporate Member of Freie Universität Berlin and Humboldt Universität zu Berlin, Berlin, Germany; ^2^Department of Linguistics, University of Potsdam, Potsdam, Germany; ^3^IPB Institut für Integrative Psychotherapieausbildung Berlin, MSB Medical School Berlin, GmbH, Berlin, Germany

**Keywords:** coherence, speech analysis, automated analysis, natural language processing, artificial intelligence, psychosis, schizophrenia, formal thought disorder

## Abstract

**Background:**

Impairments in speech production are a core symptom of non-affective psychosis (NAP). While traditional clinical ratings of patients’ speech involve a subjective human factor, modern methods of natural language processing (NLP) promise an automatic and objective way of analyzing patients’ speech. This study aimed to validate NLP methods for analyzing speech production in NAP patients.

**Methods:**

Speech samples from patients with a diagnosis of schizophrenia or schizoaffective disorder were obtained at two measurement points, 6 months apart. Out of *N* = 71 patients at T_1_, speech samples were also available for *N* = 54 patients at T_2_. Global and local models of semantic coherence as well as different word embeddings (word2vec vs. GloVe) were applied to the transcribed speech samples. They were tested and compared regarding their correlation with clinical ratings and external criteria from cross-sectional and longitudinal measurements.

**Results:**

Results did not show differences for global vs. local coherence models and found more significant correlations between word2vec models and clinically relevant outcome variables than for GloVe models. Exploratory analysis of longitudinal data did not yield significant correlation with coherence scores.

**Conclusion:**

These results indicate that natural language processing methods need to be critically validated in more studies and carefully selected before clinical application.

## 1. Introduction

Patients with non-affective psychosis (NAP), such as schizophrenia and schizoaffective disorder, can show limited function in a range of cognitive, emotional, and social abilities, including speech comprehension and speech production, affecting vocabulary, semantics, pragmatics, cohesion, and coherence (1). Incoherence, one of the most frequently examined features of speech in patients with NAP, is a type of formal thought disorder (FTD) and defined in psychiatry as the loss of meaningful associations in patients’ speech (2–4). Jaspers (5) and Bleuler (6) already described incoherence as an essential aspect of the altered self-experience in schizophrenia, which is considered a core feature of the disorder. Incoherent speech can entail severe impairments of functioning and impede societal inclusion as well as complicate therapeutic interventions (2, 7). In linguistics, discourse coherence refers to the connectedness of speech beyond the level of individual sentences, which involves topicality, reference, and thematic structure of a text (8). Discourse coherence is maintained on many different levels – intonational, lexical, syntactic, logical. It is present as local coherence, connecting sentences and their parts, as well as global coherence, as the overall topic of speech. Incoherence in the speech of patients with a NAP diagnosis can be evaluated by clinical experts, in expert-rating scales such as the Thought, Language and Communication Scale (TLC, 4), or the Scale for the Assessment of Positive Symptoms (SAPS, 9). However, clinical ratings of coherence are naturally prone to bias because they depend on the clinician’s intuition and ability to comprehend patients, i.e., on their mental representation of patients’ speech (10). In contrast, modern methods of Natural Language Processing (NLP), a field in Machine Learning, offer means of analyzing speech automatically and consistently – and they could potentially be more objective than clinical assessments. NLP methods may find subtle changes in patients’ speech that are not confounded by the listener’s interpretation of what they hear or read and potentially not detectable by human listeners, even when they are trained clinicians. This is what makes NLP and machine learning methods in general powerful: possibly detecting patterns that are not noticeable to humans, going beyond clinical impressions and making NLP methods potentially useful in early detection of psychosis onset and exacerbation.

By now, many studies have reported evidence for the association between coherence scores and patient status: Coherence scores were shown to differentiate between NAP patients or people at clinical high risk (CHR) for psychosis versus healthy controls (11–16), and predict psychosis onset in CHR populations (17, 18). Moreover, multiple studies have found that coherence measures were significantly correlated with clinical ratings of FTD in NAP patients (15, 16) and people at CHR for psychosis (19). Some researchers have suggested that incoherence may be a promising biomarker for NAP and could be linked to other biomarkers in genetic or neuroscientific research in the future (20, 21). However, the clinical relevance and therapeutic value of NLP methods in psychiatry still needs to be proven – especially against the background that a prognostic assessment may significantly affect or even stigmatize individuals (22) and that patients may be triggered by stressful situations including clinical settings (23).

Moreover, there is a need for more NLP studies with non-English speaking patients since current coherence models appear to have limited generalizability across different languages (24). In addition, studies on coherence in NAP have used a large variety of different models, embeddings and training data, raising the question which approach may promise the highest predictive value. Approaches to discourse coherence that have been successfully automated are global and local coherence as well as tangentiality (i.e., modeling how relevant a response is to a question). Elvevåg et al. were the first to introduce an automated approach to measuring global coherence and tangentiality, while the incoherence model by Bedi et al. automated local coherence. The found association between tangentiality and clinical ratings of FTD could not be replicated (16, 25, 26). Iter et al. (27) used tangentiality and local coherence methods with various embeddings and sentence-averaging methods – only four out of the 20 models tested were able to differentiate patients from controls. Similarly in another study (25), three out of 13 coherence scores showed group differences between CHR individuals and controls, and none were significant after correcting for multiple testing. To this end, NLP methods need to be further validated.

The general aim of this primarily methodological, exploratory study was to further validate NLP methods for coherence analysis in NAP, namely local and global coherence, adapted from Bedi et al. and Elvevåg et al., as well as to compare two different word embeddings [GloVe (28) vs. word2vec (29)]. We chose coherence over tangentiality since coherence scores have outperformed tangentiality in former research (16). Since coherence scores may represent different patterns in patients’ speech compared to clinical ratings, they should not only be validated against clinical ratings, but also against external criteria which are associated with functioning and psychopathology and may represent important events such as exacerbation. And as the potential clinical and therapeutic value of algorithms lies in their predictive power, we aim to include data both from cross-sectional and longitudinal measurements. If NLP methods can predict the aspects of psychotic disorders beyond clinical ratings, this could further prove their usability in psychiatry, potentially help to identify individuals at high risk and at best prevent exacerbation or hospital admission. In summary, the specific aims of this study were first, comparison of coherence scores derived from different NLP methods of coherence analysis (global vs. local), second, comparison of different word embeddings (GloVe vs. word2vec), and third, validation against clinical ratings and external criteria from cross-sectional and longitudinal measurement points. Our analysis is exploratory, aiming to pave the way for future validation studies.

## 2. Method

### 2.1. Participants

The study is based on a sub-sample of the MPP-S study (ClinicalTrials.gov-ID: NCT02576613), a randomized controlled trial called: “Modified Psychodynamic Psychotherapy for Patients with Schizophrenia.” It was conducted from December 2015 to December 2021 in cooperation with the International Psychoanalytic University (IPU) at the Charité Universitätsmedizin in Berlin, Germany – including a baseline and further measurements after 6 months, one, two, and 6 years. A sub-sample was taken from the 6 months follow-up, defined as time one (T_1_), as this was the first time that speech samples were collected in the study.

*N* = 71 patients were included with a diagnosis of schizophrenia (*n* = 51) or schizoaffective disorder (*n* = 20), according to Diagnostic and Statistical Manual of Mental Disorders, Fourth Edition, Text Revision (DSM-IV-TR, 30), confirmed by trained clinicians. For this study, transcribed speech samples were only available from T_1_. Clinical data for the sample were taken from T_1_ and, in order to include longitudinal data, from a one-year follow-up measurement after baseline, time two (T_2_). Not all patients had participated at T_2_, so that longitudinal data were available for *n* = 54 out of the initial 71 patients. Inclusion criteria beyond diagnosis were age between 18 and 65 years and native proficiency in German language. Exclusion criteria were organic brain diseases, other relevant somatic diseases, active substance dependence, or acute suicidality. Sociodemographic data and characteristics of illness are presented in Table 1. The study was approved by the ethics committee of the Charité Universitätsmedizin Berlin (approval number: EA1/200/15). All participants provided written informed consent.

**Table 1:** Characteristics of the sample.

	T_1_ (*N* = 71)	T_2_ (*N* = 54)
Age (years)	38.83 (±10.44)^a^	39.19 (±10.39)
Sex (male)	45 (63.4%)^b^	33 (61.1%)
Verbal IQ	105.35 (±12.15)	105.54 (±12.28)
Education years	15.32 (±3.42)	15.98 (±3.48)
Main diagnosis		
F20.x	51 (71.8%)	38 (70.4%)
F25.x	20 (28.2.%)	16 (29.6%)
Patients with comorbid psychiatric disorder	15 (21.1%)	12 (22.2%)
Patients with comorbid somatic disorder	29 (40.8%)	24 (44.4%)
Patients with current antipsychotic medication	62 (87.3%)	47 (87.0%)

a Mean (standard deviation).

b Frequency (percent).

### 2.2. Measures

#### 2.2.1. Natural language processing

##### 2.2.1.1. Speech samples

We used the Narrative of Emotions Task (NET, 31) to collect speech samples, a short semi-structured interview, originally developed to assess social cognition, at T_1_. We used a short version of the NET, translated into German, including three questions about four basic emotions: sadness, fear, anger, and happiness: (1) What does this emotion mean to you? (2) Describe a situation where you felt this emotion, and (3) Why do you think you felt this emotion in this situation? All interviews were conducted by trained clinicians (including SJ, A-LB, CM), recorded and manually transcribed by two authors (SJ, MS), following defined rules for transcription. Collecting speech samples from answers to (semi-)structured questions is a frequent and economic method in NLP studies (11, 15, 16, 18, 27), increases comparability, and has been shown to outperform analysis of free conversational speech (14).

##### 2.2.1.2. Annotation

The data consisted of 71 recorded and transcribed NET interviews. Transcripts underwent systematic preprocessing to reduce bias in analysis (15, 16, 27). Uniform sentence annotation guidelines were established for manual coding of sentence boundaries based on syntax, as has been done elsewhere (24). Clear annotation guidelines for sentence separation are crucial as automated coherence metrics are calculated over sentences and thus, can be influenced by sentence boundary decisions (32, 33). A sentence was defined as at least containing a subject and verb (e.g., “John eats.”). The main and the corresponding side clauses were grouped together as one sentence (e.g., “John eats when he is hungry.”). Incomplete main clauses were ended on a period (“John eats when. No, I wanted to say something else.”), main clauses connected by conjunctions were separated (“John eats when he is hungry. And he laughs when he is happy. And he sleeps when he is tired.”).

##### 2.2.1.3. Preprocessing

The interviews were split into questions on each emotion, and the questions themselves were left out of the analysis. Verbal fillers (such as “ehm”) and German stopwords were removed from the transcripts. The words were lemmatized (e. i. put to their dictionary form). The resulting transcripts had an average length of 243 words (range 57–824) with 140 unique words (range 48–358). The interviews were split into sentences using nltk.sent_tokenize.

##### 2.2.1.4. Vector-based coherence metrics

One of the key NLP methods is word embeddings. In this method, words in a text are mathematically represented as vectors. Different word embeddings utilize different methods of vectorization (see Almeida and Xexéo (34) for an overview). Semantic coherence can be approximated with a mathematical function on these vectors so that semantically similar words have vectors that are closer together (17). This definition does not try to reflect whether the discourse is intelligible but focuses on how semantically similar the words or sentences are to each other.

Word2vec (29) and GloVe (28) are traditional word embedding models which represent words as vectors and have been most widely used in NLP research with NAP patients. To ensure comparability with former research (12, 14–16, 20, 27, 35), we decided to use word2vec and GloVe models in this study. Moreover, more advanced word embeddings such as BERT and ELMo appear to yield similar coherence scores as compared to word2vec and GloVe (25).

Two open source vectorization models were used to compare their task-sensitivity. The first model used was the Spacy’s (36) tok2vec model (de_core_news_lg, specifically) trained on OSCAR Common Crawl and German Wikipedia. The second model was a GloVe model trained on German Wikipedia provided by deepset (37). The words absent from the models’ vocabularies (out-of-vocabulary words) were left out of the analysis. The word vectors were averaged across the sentence to obtain a sentence vector.

Two cosine similarity-based metrics were used to assess the coherence of the interviews. Cosine similarity is a measure of vector proximity used to assess semantic and grammatical similarity of words or sentences encoded by the vectors.

The first metric is local coherence (or first-order coherence), defined as the cosine similarities between adjacent sentences (17). This coherence metric has been the most widely used metric in NLP research with schizophrenia patients (24). The similarity is averaged across all sentence pairs in the text.


$$localcoherence\left(S\right)=\frac{\sum_{i=1}^{N-1}\cos\;sim\left(s_{i},s_{i+1}\right)}{N},$$

where *S* is a list of sentences from $s_{1}$ to $s_{N}$ .

The second metric is global coherence, defined as the cosine similarity between each sentence and the average of all sentences [adapted from Elvevåg et al. (11)]. The similarity is averaged across all sentences in the text.


$$globalcoherence\left(S\right)=\frac{\sum_{i=1}^{N-1}\cos\;sim\left(s_{i},s_{mean}\right)}{N},$$

where $s_{mean}=\frac{\sum_{\mathrm{i=1}}^{N}s_{i}}{N}$ , and *S* is a list of sentences from $s_{1}$ to $s_{N}$ .

Both metrics were calculated for each question for each participant twice, based on the two different vectorization models. The metrics were then averaged across the emotion questions to obtain four metrics of coherence per participant, two local and two global coherence scores, one for each model.

The code used for the analysis is available on request.

#### 2.2.2. Clinical measures

The expert-rated Positive and Negative Syndrome Scale was used to assess psychopathology. The scale contains 30 items, rated on a 7-point Likert scale (from 1 = absent to 7 = extreme). In further analysis, we applied a five-factor solution derived from van der Gaag et al. (38, 39) to the data, namely: positive and negative symptoms, disorganization, excitement, and emotional distress.

The MINI-ICF is a short version of the WHO International Classification of Functioning, Disability and Health where experts rate 13 subdimensions of functioning on a 5-point Likert scale (from 0 = no impairment to 4 = total disability). The rating requires a comparison between the actual and the premorbid state, so that disease-related changes in functioning are represented. A sum score was built for further analyses.

We selected external criteria of illness which are associated with functioning and psychopathology and may represent severity of illness beyond clinical ratings. External criteria we included were days of inpatient psychiatric treatment during the last 2.5 years and 0.5 years before T_1_ as well as 0.5 years after T_1_, and two characteristics of illness (age at psychosis onset, duration of illness). These variables were assessed as part of an interview regarding sociodemographic and medical characteristics.

The German vocabulary test Wortschatztest (WST, 40) was used to control for verbal IQ.

### 2.3. Statistics

Statistical analysis was performed using IBM SPSS Statistics for Windows (version 29.0, SPSS Inc., Armonk, NY, United States). Pearson correlations were computed to examine the relationship between coherence scores and continuous outcome variables at T_1_. For categorial variables (sex, antipsychotic medication), independent *t*-tests were computed to analyze mean differences in coherence scores. Partial correlations were computed to examine the relationship between the coherence scores at T_1_ and the outcome variables at T_2_ while controlling for expression of the outcome variables at T_1_. The correlational analyses are exploratory. Thus, *p*-values are only given for descriptive reasons.

## 3. Results

### 3.1. Cross-sectional data: correlations at T_1_

Table 2 shows the mean coherence scores and expression of outcome variables of participants, Table 3 shows all exploratory correlations at T_1_. The four coherence scores were highly correlated with each other. Comparison of local vs. global coherence scores did not yield divergent results. Except for one case, where the global coherence score of the GloVe model was significantly correlated with the sum score of the MINI-ICF and its local coherence score was not, all global and local coherence scores of the GloVe and word2vec model showed the same significant correlations.

**Table 2:** Coherence scores and clinical outcome variables.

	Participants’ (*N* = 71) scores at T_1_ *M* (*SD*)
Local coherence GloVe	0.77 (±0.07)
Global coherence GloVe	0.86 (±0.05)
Local coherence word2vec	0.57 (±0.07)
Global coherence word2vec	0.72 (±0.05)
PANSS positive symptoms	13.34 (±6.25)
PANSS negative symptoms	15.66 (±6.86)
PANSS disorganized symptoms	16.87 (±5.91)
PANSS excitement	12.62 (±3.97)
PANSS emotional distress	17.32 (±5.85)
Mini-ICF sum score	16.3 (±9.34)
Age at psychosis onset	25.36 (±7.63)
Duration of illness (years)	13.47 (±9.05)
Days of inpatient care 2.5 years before T_1_	39.84 (±68.43)
Days of inpatient care 6 months before T_1_	10.07 (±34.39)
Days of inpatient care 6 months after T_1_^a^	5.74 (±16.16)

PANSS, Positive and Negative Syndrome Scale, five-factor solution of van der Gaag et al. (38, 39); MINI-ICF, International Classification of Functioning, Disability and Health (short version) (41).

a Days of inpatient care 6 months after T_1_ was assessed at T_2_ with *N* = 54 patients.

**Table 3:** Exploratory correlational analysis: Pearson correlations between coherence scores and clinical outcome variables at T_1_ (*N* = 71).

	Local coherence GloVe	Global coherence GloVe	Local coherence word2vec	Global coherence word2vec
Local coherence GloVe	–	0.969^**^	0.576^**^	0.487^**^
Global coherence GloVe	0.969^**^	–	0.569^**^	0.533^**^
Local coherence word2vec	0.576^**^	0.569^**^	–	0.923^**^
Global coherence word2vec	0.487^**^	0.533^**^	0.923^**^	–
Duration of illness	0.087	0.059	0.056	−0.012
Age at psychosis onset	0.037	0.128	−0.090	0.008
Days of inpatient care 2.5 years before T_1_	−0.163	−0.165	−0.078	−0.094
Days of inpatient care 6 months before T_1_	−0.190	−0.193	−0.253^*^	−0.257^*^
Days of inpatient care 6 months after T_1_	−0.081	−0.107	0.107	0.117
PANSS positive symptoms	−0.018	−0.047	−0.153	−0.223
PANSS negative symptoms	−0.279^*^	−0.269^*^	−0.310^**^	−0.264^*^
PANSS disorganized symptoms	−0.175	−0.199	−0.311^**^	−0.359^**^
PANSS excitement	−0.054	−0.093	−0.278^*^	−0.331^**^
PANSS emotional distress	−0.135	−0.143	−0.129	−0.160
Mini-ICF sum score	−0.227	−0.266^*^	−0.164	−0.209

PANSS, Positive and Negative Syndrome Scale, five-factor solution of van der Gaag and colleagues (38); MINI-ICF, International Classification of Functioning, Disability and Health (short version) (41). *p*-values are reported for descriptive reasons: ^*^p < 0.05; ^**^p < 0.01.

Examining correlations of the GloVe and word2vec model showed that the GloVe model yielded three significant correlations with outcome variables while there were eight significant correlations with the word2vec model.

Regarding exploratory validation against clinical ratings, all coherence scores showed significant negative correlations with the PANSS factor for negative symptoms. In addition, local and global coherence scores of the word2vec model showed significant negative correlations with the PANSS factor for disorganized symptoms and excitement. None of the four coherence scores were significantly correlated with the PANSS factors for positive symptoms and emotional distress. Global coherence scores of the GloVe model were significantly negatively correlated with the sum score of the MINI-ICF.

Regarding external criteria, there was a significant negative correlation between local and global coherence scores of the word2vec model and the days in inpatient treatment 6 months before T_1_. There was no significant correlation between coherence scores and days in inpatient treatment 2.5 before or 0.5 years after T_1_ or characteristics of illness (age at psychosis onset, duration of illness).

When controlling for psychopathology in a partial correlation by including the five PANSS factors as control, the correlations with the MINI-ICF and days of inpatient care did not remain significant.

Coherence scores were not significantly correlated with the control variables age, education years, verbal IQ, length of transcripts in words, nor did they differ significantly between patients with schizophrenia or schizoaffective disorder, or between patients who did or did not take antipsychotic medication. However, the global coherence score of the GloVe model was significantly negatively correlated with transcript length. Also, female patients’ coherence scores of the global word2vec model were significantly higher than those of men. Closer examination of sex differences in the sample through independent *t*-tests revealed that men had significantly higher mean values in four PANNS factors as compared to women: positive and negative symptoms, disorganization, excitement.

### 3.2. Longitudinal data: correlations at T_2_

None of the partial correlations between coherence scores at T_1_ and outcome variables at T_2_ were significant. The partial correlations as well as Pearson correlations between all variables at T_1_ and T_2_ are provided in the Supplementary Tables S1, S2.

## 4. Discussion

The overall aim of this study was an exploratory analysis of correlations between coherence scores and clinical outcome variables to carve out a potential direction for future NLP validation studies. The specific aims of the study were, first, to compare different NLP methods of coherence analysis, second, to compare different word embeddings, and third, to validate them against clinical ratings and external criteria – using both cross-sectional and longitudinal clinical data.

Comparison of different NLP methods and word embeddings revealed that the word2vec models (both global and local coherence) were significantly correlated with four clinical outcome variables while the GloVe models were significantly associated with only one and two outcomes, respectively. The global GloVe model was also sensitive to transcript length – a potential confounding factor in its coherence scores. Furthermore, coherence scores generated with the global word2vec model showed significant sex differences. One could argue that the global word2vec model was the only model to represent that male patients had significantly higher ratings of psychopathology than female patients in this sample. While these results should be interpreted with caution, it might imply that the word2vec models outperformed the GloVe models in calculating coherence scores that were associated with clinically relevant outcomes in this study. This corresponds to findings by Iter et al. (27) who found significant group differences between patients and controls for the word2vec incoherence model, not GloVe, but contrasts our own previous study that found a superiority of the GloVe model in prediction of psychopathology in NAP (16). These results indicate that the choice of NLP model should not be arbitrary. It has to be taken into account that different models, that is models with different architecture (e.g., GloVe vs. word2vec) as well as models trained on different corpora, produce different word vectors – this could explain the different results between this current and the previous study (16), having used different training data as well as different preprocessing and a more sophisticated sentence annotation. A concern would be that the chosen model has a stronger effect on the coherence scores than the difference between groups or coherence metric used (e.g., local vs. global). In this study, all coherence scores were still highly correlated with each other (see Table 2). The reason for this is, probably, the fact that the two models are both trained on the same material, that is German Wikipedia. If models are trained on different material, correlation between them can be low. This might be one of the key challenges of cross-linguistic application of NLP methods (24) and the reason for limited replicability of the results within one language across models (25) and studies (16, 25, 26). Nevertheless, our results show that models trained on the same material can still yield different results. The effect of different embedding models both intra- and cross-linguistically therefore requires further investigation.

It should be noted that the number of significant correlation coefficients should not be the only criterion for choosing a model. As Holmlund et al. (42) put it, “There is no ‘one size fits all’ approach to choosing the right operationalization of disorganization in speech” (p. 3). While there is more need for validation studies of different embeddings, future studies should also aim to understand better which coherence metrics represent which specific impairments in patients’ speech (42).

Concerning validation against clinical ratings, one might expect to find most reliable correlations between coherence scores and ratings of incoherence, in this study represented in the PANSS factor for disorganization. After all, the embeddings are supposed to model speech (in)coherence and have been found to be correlated with ratings of positive FTD and incoherence in former studies (15, 16, 19). However, we found the most consistent correlation between coherence scores and the PANSS factor for negative symptoms. A significant correlation with the factor for disorganization was only found for the word2vec models. This may support the assumption that word2vec models outperformed GloVe models in our sample. From an application perspective, other questions arise: On the one hand, one could argue that coherence scores could be useful as long as they are correlated with any relevant characteristics of the illness – positive FTD or not. Identification and treatment of psychosis may sometimes focus too much on productive symptomatology. Clinical application and usefulness of NLP methods would rely on their ability to predict illness – our results suggest that we should focus more on negative symptoms in this context. These often represent the first sign of the onset of psychosis and occur before the onset of positive symptoms (43). If NLP coherence scores are significantly correlated with clinical appearance of negative symptoms, this might explain their effectiveness in prediction of psychosis onset (17, 18). On the other hand, not all characteristics of illness are a sign of psychosis onset or exacerbation and require urgent intervention. As mentioned in the introduction, early labeling can also lead to stigmatization (22). While negative symptoms may be a predictor of psychosis onset, they are not a sign of acute exacerbation characterized more by positive symptoms. One may conclude that correlation with clinical ratings of psychopathology appears to be a necessary but not sufficient condition for the predictive power of NLP coherence models in NAP. Their application may also depend on the prediction of subtle changes in patients’ life that are not recognized easily or early enough by clinicians for appropriate intervention.

To this end, we attempted an exploratory validation of NLP methods against external criteria and longitudinal data. This analysis revealed only one significant correlation between the word2vec models and days of inpatient care 6 months before the speech samples were collected. However, this correlation did not remain significant after controlling for psychopathology. This result may question the potential usefulness of NLP coherence analysis to predict relapse. If NLP analyses cannot outperform clinical ratings to predict exacerbation, a clinically relevant benefit for patients seems uncertain. On the other hand, NLP methods have been proven powerful in the prediction of psychosis onset in the past (17, 18). Also, this is the first study to validate coherence scores against external criteria and longitudinal data. Selection of variables might not have been appropriate to operationalize exacerbation beyond clinical ratings. We recommend further attempts to validate NLP methods against external and longitudinal data in future research.

### 4.1. Limitations

There are some limitations that should be taken into account. As results did not have any predictive value with respect to outcomes, future research should include “harder” external criteria and more data on patients’ course of illness. For instance, medical health records about hospitalization and medication could be examined to utilize more objective data on exacerbation. As mentioned above, the consequences and risks of stigmatization due to false predictions for the individual should always be critically reflected upon and results interpreted with appropriate caution. The results should be replicated with larger sample sizes, other diagnostic groups, healthy controls as well as CHR individuals. Moreover, in this study we decided to focus solely on NLP coherence scores. Past NLP research has shown that inclusion of other characteristics of patients’ speech can improve predictive value of coherence models (15, 17, 18, 27, 35) – for instance, syntactic features, referential ambiguities, neologisms, cohesion scores, perseverations, and acoustic features. Since different patterns of linguistic impairment appear to be associated with different levels of psychopathology and functioning (44), future studies should consider developing and validating speech models of NAP containing more features of speech than coherence. Psychometric evaluation of coherence scores should also examine reliability, e.g., by intra-individual correlation of coherence scores across multiple measurement time points.

A statistical limitation is the large number of different output parameters, which could have led to an overestimation of significance.

### 4.2. Implications and conclusion

The study showed that coherence scores derived with NLP methods are correlated with clinical ratings of psychopathology, but not with external or longitudinal data. The word2vec model was significantly correlated with more variables than the GloVe model while there were no major differences between local and global coherence models. While the results support construct validity of NLP models of coherence, they raise questions about the usefulness of their application in the clinical context. As results for the used models differed, we recommend careful selection of model and training data.

It remains an important task for clinical researchers to engage in the debates and studies revolving around machine learning in psychiatry. Clinical experts’ considerations about the ethics, feasibility and usefulness of machine learning and NLP methods in the field need to always accompany this research.

## Data availability statement

The raw data supporting the conclusions of this article will be made available by the authors, without undue reservation.

## Ethics statement

The studies involving human participants were reviewed and approved by Ethikkommission der Charité – Universitätsmedizin Berlin. The patients/participants provided their written informed consent to participate in this study.

## Author contributions

SJ and A-LB contributed equally to the manuscript by developing and testing the hypotheses and writing the first draft. GR conducted the NLP analysis and made an essential contribution to the draft. MS completed the annotation of all transcripts. CM was responsible for and conceptually involved in the conduct of the study. SJ, A-LB, GR, IN, MS, FB, AH, and CM critically read and revised the manuscript. All authors contributed to the article and approved the submitted version.

## Funding

CM received personal funding (Berlin Institute of Health BIH_PRO_279). The MPP-S study is supported by Deutsche Gesellschaft für Psychoanalyse, Psychotherapie, Psychosomatik und Tiefenpsychologie (DGPT e.V.) and by Köhler Stiftung (Grant number: S112/10211/16). We acknowledge financial support from the Open Access Publication Fund of Charité – Universitätsmedizin Berlin and the German Research Foundation (DFG).

## Conflict of interest

The authors declare that the research was conducted in the absence of any commercial or financial relationships that could be construed as a potential conflict of interest.

## Publisher’s note

All claims expressed in this article are solely those of the authors and do not necessarily represent those of their affiliated organizations, or those of the publisher, the editors and the reviewers. Any product that may be evaluated in this article, or claim that may be made by its manufacturer, is not guaranteed or endorsed by the publisher.
