# Supplementary material for: Validation of natural language processing methods capturing semantic incoherence in the speech of patients with non-affective psychosis
Source: Front Psychiatry. 2023 Jul 25;14:1208856. doi: 10.3389/fpsyt.2023.1208856 (PMC10411549; doi:10.3389/fpsyt.2023.1208856)
Supplement: Supplementary file 1 [file Table_1.docx]

Supplementary Material

Validation of natural language processing methods capturing semantic incoherence in the speech of patients with non-affective psychosis

Sandra Anna Just †^1*^, Anna-Lena Bröcker †^1^, Galina Ryazanskaya^2^, Ivan Nenchev^1^, Maria Schneider^3^, Felix Bermpohl^1^, Andreas Heinz^1^, Christiane Montag^1^

†These authors contributed equally to this work and share first authorship.

*** Correspondence:**Sandra Anna Just
sandra-anna.just@charite.de

# Supplementary Tables

| **Table S1**  Exploratory correlational analysis: Partial correlations between coherence scores and clinical outcomes at T_2_, controlling for their expression at T_1_ (*N* = 54). | | | | |
| --- | --- | --- | --- | --- |
|  | local coherence GloVe | global coherence GloVe | local coherence word2vec | global coherence word2vec |
| Days of inpatient care 6 months after T_1_ | -.006 | -.025 | .197 | .202 |
| PANSS positive symptoms | -.136 | -.151 | -.229 | -.250 |
| PANSS negative symptoms | .168 | .113 | .218 | .173 |
| PANSS disorganized symptoms | -.195 | -.256 | -.103 | -.195 |
| PANSS excitement | .018 | -.038 | -.049 | -.058 |
| PANSS emotional distress | -.204 | -.232 | -.151 | -.152 |
| Mini-ICF sum score | .090 | .120 | -.073 | -.115 |
| PANSS = Positive and Negative Syndrome Scale, five-factor solution of van der Gaag and colleagues (2006); MINI-ICF = International Classification of Functioning, Disability and Health (short version).  Partial correlations were controlled for days of inpatient care before T_1_, expression of PANSS factors at T_1_, Mini-ICF sum score at T_1_. *p*-values are reported for descriptive reasons: ^*^*p* < .05; ^**^*p* < .01. | | | | |
